# Clinical Assessment of Dizzy Patients: The Necessity and Role of Diagnostic Tests

**DOI:** 10.5539/gjhs.v6n3p194

**Published:** 2014-03-24

**Authors:** Mahsa Bakhit, Alireza Heidarian, Sara Ehsani, Maryam Delphi, Seyed M. Latifi

**Affiliations:** 1 Musculoskeletal Rehabilitation Research Center, Ahvaz Jundishapur University of Medical Sciences, Ahvaz, Iran; 2 Department of Audiology, School of Rehabilitation, Ahvaz Jundishapur University of Medical Sciences, Ahvaz, Iran; 3 Emergency Department, Apadana Hospital, Ahvaz, Iran; 4 Department of Radiology, Golestan Hospital, Ahvaz Jundishapur University of Medical Sciences, Ahvaz, Iran; 5 Diabetic Research Center, Ahvaz Jundishapur University of Medical Sciences, Ahvaz, Iran

**Keywords:** vertigo, dizziness, electrocardiography, magnetic resonance imaging, videonystagmography

## Abstract

Over administration of diagnostic tests in health care settings is a critical issue, imposing a great deal of expenditure on health sector. Vertigo and dizziness are common complaints of many patients who seek medical advice, and the vast majority of them undergo several evaluations, including Brain Magnetic Resonance Imaging (MRI), Laboratory tests, Pure Tone Audiometry (PTA), and Electrocardiography (ECG). The aim of this study was to investigate the performing rate of these diagnostic tests, and to evaluate their necessity and medical indications. This study was conducted on 270 dizzy patients referred to Apadana Dizziness and Vertigo Clinic, Ahvaz, Iran, from July 2008 to February 2013. Of these, 71.9% were diagnosed with peripheral lesions while laboratory assessment (58.1%) and brain MRI (38.1%) were the most requested tests. Age was an important factor, affecting the frequency of performing the ECG and Brain MRI. Medications were still administered widely even to those who seemed to respond well enough to vestibular rehabilitation. These findings revealed that many unnecessary and time-consuming diagnostic tests were performed, which had minor contribution to the final diagnosis and treatment of the patients. Therefore, a modification in the assessment methods of the dizzy patients with emphasis on history and clinical presentation seems essential.

## 1. Introduction

Dizziness is a rather vague and unspecified term used by patients to describe the sense of unawareness of the surroundings (Froehling, Silverstein, Mohr, & Beatty, 1994; Katsarkas, 2008), which is divided into three categories: Vertigo, Disequilibrium, and Syncope. Patients developing vertigo usually have a sense of forward-backward movement and rotation (Baloh, 1998b; Saccomano, 2012). On the other hand, disequilibrium is the patient’s inability to keep balance of the torso and is not necessarily accompanied by the sense of movement (Cohen, 1991). As for syncope, loss of consciousness, tonic-clonic movements, and a history of death in any family members before the age of 30 differentiate it from vertigo and disequilibrium (Saccomano, 2012). Peripheral lesions and psychiatric problems account for more than 70% of the causes of dizziness (Kroenke, Hoffman, & Einstadter, 2000), and laboratory tests such as evaluation of lipid profile and brain computerized tomographic scan have been found unjustifiable (Polensek & Tusa, 2009; Torres-Castro, Hendauss-Waked, Baena-Rivero, & Granados-Gomez, 2011). Furthermore, most cases of dizziness can be easily differentiated by a thorough history taking and physical examination (Saccomano, 2012). However, dizzy patients are usually visited by different specialists and undergo a variety of rather expensive evaluations in order to find a solution to their problem. This research tries to investigate the different types of medical evaluations usually applied for the dizzy patients, and to evaluate the request rate, necessity and medical indications for these diagnostic tests.

## 2. Materials and Methods

This study was conducted on the medical records and clinical findings of the 270 dizzy patients referred to Apadana Dizziness and Vertigo Clinic, Ahvaz, Iran from July 2008 to February 2013. Initially, the basic assessments including taking a history and otoscopic examination were conducted for all the patients. In the History form, there was a section allotted to the evaluations previously performed for the patient, including Laboratory assessments, Brain Magnetic Resonance Imaging (MRI), Electrocardiography (ECG), and Pure Tone Audiometry (PTA). Patients were asked if any anti-vertigo medication had been administered to them, and what specialist had referred them to the clinic. Video Nystagmography (VNG) test was conducted for each patient, using Video Nystagmography Chartr system manufactured by ICS Corporation, the United States of America and Hortmann GN-Otometrics Airmattic II made by Otometrics Corporation, Denmark for cold and warm air stimulation in the Bitheraml Calorics test. Oculomotor tests (Random Saccade, Sinusoidal Smooth Pursuit, Gaze and Spontaneous Nystagmus), Positioning (Dix-Hallpike maneuver), Positional and Bithermal Caloric tests (24°C and 50°C for cold and warm air stimulation respectively) were also performed. Based on the history, physical examination performed by our otolaryngologists, and VNG test results, the final diagnoses were defined and categorized in five groups: Benign Paroxysmal Positional Vertigo (BPPV), Ménière’s Disease (MD), Central Nervous System (CNS) Lesions, Other Peripheral Lesions, and Inconclusive results. The diagnostic criteria for these pathologies were as following:

### 2.1 Diagnostic Criteria

#### 2.1.1 BPPV

A history of at least 3 attacks of rotational vertigo lasting for less than a minute, provoked by head position changes or rolling in bed, fatigable nystagmus with vertigo in Dix-Hallpike maneuver, and the absence of any signs and symptoms of a central lesion (Polensek & Tusa, 2009).

#### 2.1.2 Ménière’s Disease

Fluctuating hearing loss, a history of episodic rotational vertigo lasting for minutes associated with nausea and vomiting, aural fullness, perspiration, tinnitus, the absence of head trauma, and the elevated SP/AP index in the Electrocochleography (EcochG) test (SP/AP > 0.5) (Devaiah, Dawson, Ferraro, & Ator, 2003).

#### 2.1.3 Central Lesions

The patient’s inability of standing without support, direction changing nystagmus in a fixed position, persistent spontaneous nystagmus, and pure horizontal, torsional or vertical spontaneous nystagmus with changes in direction as the gaze changes. Other neurological signs such as facial numbness, lack of coordination, and suspicious findings in oculomotor tests (Baloh, 1998a, 1998b).

#### 2.1.4 Other Peripheral Lesions

Unilateral/Bilateral Caloric Weakness or Directional Preponderance, severe nausea and vomiting, normal oculomotor results and lack of any neurologic symptoms such as numbness and direction changing nystagmus (Baloh, 1998b).

#### 2.1.5 Inconclusive results: Normal Findings in all the Tests without any Neurological Symptoms

The results were statistically assessed with SPSS software version19, and the Chi-Square test and independent T-Test was performed to analyze them.

## 3. Results

Two hundred and seventy patients were evaluated in our clinic of which 58.4% were female, and 45.20 % were male. Mean age was at 49.48±12.16, with the minimum and maximum being 21 and 86 years respectively. The patients were divided into 3 subgroups based on their age: 21 to 40, 41 to 60, and 61 to 86 years old. 54.10 % of the patients were 41 to 60 year old, and the age category of 61 to 86 year olds had the least population (19.60%). Most patients were referred to the clinic by the ENT specialists (121 patients – 44.80%) and Emergency Department (ED) practitioners (94 patients – 34.80%). Neurologists, Cardiologists, and other specialists referred 13% (35 patients), 1.9% (5 patients), and 5.6% (15 patients) respectively.

Among the diagnostic modalities, laboratory tests had the highest rate of request by physicians, performed in 58% of all the patients in which 84% had the normal results while the ECG was ordered in only 14.1%. Medications including Betahistine hydrochloride and Dimenhydrinate were administered to 83.7% of the patients. The Brain MRI and PTA were requested in 103(38.14%) and 57(21.11%) patients respectively. 77 patients (28.5%) suffered from BPPV, whereas 28(10.40%) were finally diagnosed with Ménière’s disease. 44 patients (16.3%) diagnosed with CNS lesions were referred to neurologists, and 89 patients (33%) had other peripheral lesions. Furthermore, in 32 patients (11.85%), the results were inconclusive. Tables 1 and Table 2 show the diagnostic findings of all the patients.

**Table 1:** Distribution of the specialities who referred the patients by different pathologies

	BPPV n=77(28.5%)	Ménière’s n=28(10.4%)	Other Peripheral n=89(33%)	CNS n=44(16.3%)	Inconclusive n=32(11.85%)
ENT	33(42.9%)	15(53.6%)	41(46.1%)	18(40.9%)	14(43.8%)
ED	31(40.3%)	8(28.6%)	29(32.6%)	16(36.4%)	10(31.3%)
Neuro.	8(10.4%)	2(7.1%)	16(18%)	5(11.4%)	4(12.5%)
Cardio.	2(2.6%)	0(0.0%)	1(1.1%)	1(2.3%)	1(3.1%)
Other	3(3.9%)	3(10.7%)	2(2.2%)	4(9.1%)	3(9.4%)
Total	77(100%)	28(100%)	89(100%)	44(100%)	32(100%)

**Table 2:** Distribution of diagnostic tests in different pathologies

	MRI n=103(%)	PTA n=57(%)	ECG (%) n=38	Laboratory Tests n=157(%)	Medication n=226(%)
BPPV	27(26.21)	18(31.35)	11(28.94)	41(26.11)	61(26.99)
Ménière	12(11.65)	3(5.26)	7(18.42)	17(10.82)	23(10.17)
CNS	22(21.35)	9(15.78)	4(10.52)	21(13.37)	41(18.14)
Other Peripheral	28(27.18)	20(35.08)	12(31.57)	58(36.94)	75(33.18)
Inconclusive	14(13.59)	7(12.28)	4(10.52)	20(12.73)	26(11.50)
Total	103(100)	57(100)	38(100)	157(100)	226(100)

Pearson Chi-Square test revealed that the frequency of the performing Brain MRI and ECG, was significantly higher in the age group of 61 to 86 year olds than the other two age groups (p<0.001), but this was not the case in Pure Tone Audiometry (Table 3). On the other hand, the independent T-Test indicated that there was no relation between the final diagnosis and sex, the sex and the number of requested tests, and different age groups and the final diagnosis (p>0.05).

**Table 3:** Comparison of request rate for brain MRI, PTA, and ECG in different age groups

Age	MRI	PTA	ECG	Total
21-40	23(32.39%)	16(22.22%)	4(5.55%)	71(100%)
41-60	48(32.87%)	34(23.28%)	7(4.79%)	146(100%)
61-86	32(60.37%)*	7(13.20%)	27(50.94%)*	53(100%)
Total	103(100%)	57(100%)	38(100%)	

* p value< 0.05.

## 4. Discussion

The findings showed that 71.90% of our cases suffered from peripheral lesions, 11.85% were inconclusive, and in 16.30%, there were evidence of central lesions. These findings were similar to those of Kroenke and colleagues (2000). Laboratory tests, Brain MRI, PTA, and ECG were commonly ordered by physicians prior to our evaluation at the Vertigo and Dizziness clinic. Polensek and Tusa (2009) also found that these tests along with Brain CT-Scan and MRI with angiography (MRA) were requested frequently for their BPPV patients.

Like another survey (Vannucchi, Pecci, & Giannoni, 2012), 28.5% of our patients were diagnosed with BPPV of which 35.1% had an MRI imaging done, but it has been proved that it is of no diagnostic value in BPPV (Polensek & Tusa, 2009). Regarding the central lesions resembling BPPV, a proper differentiation can be reached by focusing on the symptoms and signs of these lesions. Unconventional nystagmus in Dix-Hallpike, resistance to the usual rehabilitation, hearing loss, tinnitus and spontaneous nystagmus are some of these symptoms, which are not usually seen in BPPV (Herdman, Blatt, & Schubert, 2000). Dix-Hallpike is a simple diagnostic maneuver introduced in 1952, and is one of the most effective methods to diagnose BPPV, yet it is not usually practiced by most physicians. Vestibular rehabilitation, on the other hand, is the best treatment for BPPV (Fujino et al., 1994; Vaz Garcia, 2005), yet anti-vertigo medications including Dimenhydrinate, Betahistine hydrochloride and Diazepam (Valium) were administered to the 79.2% of our BPPV patients as their first choice of treatment.

Brain MRI was applied for 38.1% of our patients, and in 65% of them, the results was normal. Literatures show that peripheral and psychiatric etiologies, account for more than 70% of all vertigo cases. Vertigo and disequilibrium are rare signs of skull base neoplasms, and in most cases, these neoplasms are accompanied by other neurological signs and symptoms, including facial numbness, headaches and diplopia (Herdman et al., 2000; Marzo & Leonetti, 2000). Literatures urge physicians to order a Brain MRI more in the patients presenting with sustained vertigo for at least 6 months (Marzo & Leonetti, 2000). An effective bed-side method to differentiate peripheral and central lesions is Head Thrust Test (HTT), which is fast to perform, and requires no special equipment. Patient’s head is quickly turned to one side and then to the other side in about 10º. The observation of corrective saccades in this test reveals a deficiency in Vestibulo-Ocular Reflex (VOR), which is a sign of peripheral lesions. The Central pathologies on the other hand, leave the VOR intact, and no corrective (catch-up) saccades will be observed (Baloh, 2004). By using this method it is possible to come to a general decision about whether to perform a Brain MRI or not.

PTA was performed in 21.1% of the patients. The presence of hearing loss in purulent labyrinthitis and perilymphatic fistula plays a critical role in differentiating these two lesions from vestibular neuritis (Lee, 2012). Moreover, hearing loss along with tinnitus are some of the main features of Ménière’s disease (Perez-Fernandez, Montes-Jovellar, Cervera-Paz, & Domenech-Vadillo, 2010). Considering the high incidence of peripheral vestibular lesions (Kroenke et al.,2000), performing PTA in the only 21.1% of the patients seems like negligence in the diagnostic process of the giddy patients.

Despite the fact that the laboratory tests were the most commonly ordered investigation, 84% of the results were unremarkable. Most research show that metabolic disorders such as diabetes mellitus and high blood pressure affect the patients suffering from vertigo, hearing loss and tinnitus; however, dizziness is rarely seen in hyperlipoproteinemia or other lipid metabolism disorders (Kazmierczak & Doroszewska, 2001). Similar to our findings, a retrospective study of the medical records of 201 vertigo patients unveiled that metabolic evaluations including lipid profile had been performed in 76% of patients in which 80% of them had normal results (Torres-Castro et al.,2011). In an investigation of 2,332 patients with progressive hearing loss, tinnitus and vertigo, hyperlipoproteinemia was found in the only 5.1% of the patients and that most of them had already been diagnosed with other metabolic disorders including diabetes (Pulec, Pulec, & Mendoza, 1997).

ECG and Brain MRI were performed significantly more in the age group of 61-84 year olds (p<0.001). In elderlies, myocardial infarctions are often presented with unconventional symptoms such as headaches, vertigo, malaise and tinnitus. Vertigo is more common in frontal myocardial infarctions (Culic, Miric, & Eterovic, 2001), and symptoms of Lateral Medullary, Lateral Pontine and Inferior Cerebellar Infarctions are similar to Acute Peripheral Vestibular Lesions, such as vestibular neuritis and labyrinthitis (Delaney, 2003). In contrast, the risk of vascular disorders in the elder patients is higher than the others (Baloh, 2004).

## 5. Conclusion

The findings of this research more and more reveal that currently, the health care settings and medical staffs have a significant tendency towards administration of diagnostic tools, even when there is no scientific basis for them. The prevalence of peripheral vertigo is considerably higher than centrally-evoked vertigo; therefore, strong indications should exist to implement most of the diagnostic tests such as Brain MRI and CT-Scans. In fact, performing these evaluations should be preserved for those whose symptoms point to a central lesion, or their history calls for further investigation at the level of the brain. However, when it comes to the geriatrics, even in the absence of central signs, performing a Brain MRI and ECG is required to rule out infarctions. Regarding the laboratory assessments, these tests are of limited value, and do not contribute significantly in diagnosing the nature of vertigo.

The proper diagnosis of the underlying cause of vertigo is the key to successful treatment and effective rehabilitative programs. Through investing more time familiarizing ourselves with symptoms, signs and scientific facts around various pathologies, and by taking a comprehensive history and bed-side physical examinations prior to administration of sophisticated tests, we can contribute to a better approach to diagnosis and treatment of the dizzy patients, and reduce the number of unnecessary evaluations. On balance, to avoid waste of time, money, resources, and to reach a more precise diagnosis, a modification in the approach to assessment of the dizzy patients seems so imperative.
